# The Effects of Lavandula Angustifolia Mill Infusion on Depression in Patients Using Citalopram: A comparison Study

**DOI:** 10.5812/ircmj.4173

**Published:** 2013-08-05

**Authors:** Masoud Nikfarjam, Neda Parvin, Naziheh Assarzadegan, Shabnam Asghari

**Affiliations:** 1 Department of Psychiatry, Cellular and Molecular Research Centre, Shahrekord University of Medical Sciences, Shahrekord, IR Iran; 2 Herbal Research Center, Shahrekord University of Medical Sciences, Shahrekord, IR Iran; 3 Iranian Physician, Toronto, Onatario, Canada; 4 Department of Family Medicine Faculty of Medicine, Memorial university of Newfoundland, Canada

**Keywords:** Depression, Lavandula, Citalopram

## Abstract

**Background:**

Many herbs have been used to treat psychiatric disorders including anxiety and depression in traditional medicine.

**Objectives:**

This study was carried out to determine the effect of using Lavandula angustifilia infusion on depression in patients taking Citalopram.

**Patients and Methods:**

Among all patients referred to the Hajar Hospital psychiatric clinic, Shahrekord, Iran, 80 patients who met the criteria of major depression according to the structured interviews and the Hamilton questionnaire for Depression were included in the study. They were randomly assigned into two groups of experimental treatment group and standard treatment group at this study. In standard treatment group, the patients were given Citalopram 20 mg. In experimental treatment group, the patients took 2 cups of the infusion of 5 g dried Lavandula angustifilia in addition to tablet Citalopram 20 mg twice a day. The patients were followed up for four and eight weeks of the study onset using Hamilton Scale questionnaire and treatment side effects form. Data were analyzed using student t-test, pair t-test and chi square.

**Results:**

After four weeks of the trial onset, the mean depression score according to the Hamilton Scale for Depression was 17.5 ± 3.5 in the standard treatment group and 15.2 ± 3.6 in the experimental treatment group (P < 0.05). After eight weeks, it was 16.8±4.6 and 14.8±4 respectively (P < 0.01). In addition, the most commonly observed adverse effects were nausea (12.8 %) and confusion (10%). In terms of side effects, there were no significant differences between two groups.

**Conclusions:**

Considering the results of this study, Lavandula angustifilia infusion has some positive therapeutic effects on depressed patients most importantly decreases mean depression score and might be used alone or as an adjunct to other anti-depressant drugs.

## 1. Background

Depression is a major health problem in modern society and is considered as one of the most common chronic disorders (1). It can cause a significant reduction in patient’s functioning in a wide range including career, familial and social relations and may also lead to anhedonia, emotional and psychological stress(2). No single etiology has been found for depression yet, but a combination of genetical, biochemical, environmental and psychological factors are considered to have roles. Norepinephrine and Serotonin are the two neurotransmitters, which mostly involve in pathophysiology of mood disorders. Because of the significant effect of Selective Serotonin Reuptake Inhibitors (SSRIs) in treatment of depression, today, serotonin is well known as a neurotransmitter of biological amine type which is most of all related to depression(2).

Depression is a common yet serious disorder and most cases will eventually need treatment to cure (3). Although a large number of drugs can be used to treat depression, most of them may cause multiple adverse reactions including anticholinergic reactions, orthostatic hypotension, and arrhythmia. Therefore, investigation for effective drugs with minimum side effects is significantly important. However, the goal of treatment in depression is to maximize the efficacy and minimize the adverse conditions as well as maintaining a stable and satisfying state for the patient (3).

Citalopram is a selective serotonine reuptake inhibitor SSRI which is administered for treatment of depression with the dose of 20­60 mg per day(4) On the other hand, benzodiazepines are used as adjunct drugs for treatment of anxiety and insomnia disorders in 30 to 60 percept of depressed patients and their concomitant use with antidepressant agents leads to a better response and decreases the incidence of treatment discontinues in these patients; however, this treatment must be used with caution because of the possibility of drug addiction (4).

Although many antidepressant drugs are available, some psychologists believe that patients compliance is affected by numerous intolerable side effects. However, herbal medicines have less complication and might be administered alone or as a complementary therapy in these patients (5). The efficacy of some herbal remedies has been already proved in the treatment of depression such as *Hypericum perforatum* (6). Moreover, *Lavandula angustifilia* from Lamiaceae family has also been of a great attention in traditional and herbal medicine for treatment of depression (3). This herb is also used in cosmetic products for its fragrance where the flower is the main part of the plant being used in herbal remedies. The essence of this herb contains 1­3% of monoterpenes and the most important chemical constituents are linalyl acetate (30­55%), linalool (20­35%), beta­ocimene, cineole, camphor, sesquiterpene caryophyllene oxide, tonen, resmarinic acid, coumarin, and flavnoids (7). Lavandula is known to be effective in gastric disorders, headaches, particularly in tension headache. This herb has some antispasmodic, analgesic and relaxant properties (3). Likewise, clinical trials have shown that it is beneficial in treatment of sleep disorders and anxiety (8, 9).

Despite the fact that lots of herbs can be used in the treatment of a wide variety of diseases, there are few scientific documents to confirm or reinforce these experiences. Although Lavandula is mentioned in some medicinal plant textbooks to have anti-depressant characteristics, to our knowledge no supported document for this claim has been published. Of the studies conducted in this topic, we may refer to Akhundzade 2003, research on the effect of using Lavandula Angustifilia Mill Tincture and imipramine on depression in which the efficacy of their concurrent use was proved (P < 0.001) (3). Since Citalopram has some side effects including anxiety and nervousness in patients, it is usually ordered along with a sedative at the beginning.

## 2. Objectives

Considering the growing popularity of using herbal remedies among our patients and the fact that Lavandula has antidepressant and relaxant properties, we performed this study in order to determine the efficacy of the oral administration in the form of infusion on depression in depressed patients using Citalopram and compare the results with the standard treatment group who used only Citalopram.

## 3. Patients and Methods

### 3.1. Ethic

This study was carried out as a randomized clinical trial (RCT) after being approved by Ethics Committee of Shahrekord University of Medical Sciences and registered in the clinical trials website of Iran Ministry of Health.

### 3.2. Study Setting

The study was performed at the psychiatry clinic of Shahrekord University of medical sciences, Shahrekord, Iran, from February 2007 to January 2009.This clinic is the only psychiatric clinic in Shahrekord, Shahrekord is the capital city of Chaharmahal and Bakhtiari province of Iran with 127395 population in 2004.

### 3.3. Study Design and Patients

Adults aged 20 or older newly diagnosed with depression referring to psychiatry clinic of Shahrekord University Hospital. Hamilton Rating Scale for Depression was used before intervention, 4 weeks and 8 weeks after beginning of the study which consists of twenty-four questions and has been already used in many other researches. Hamilton Rating Scale for Depression has been validated in several studies (3). Patients with a total score less than 17 were considered healthy and automatically excluded from the study; those with 17-24 score were known to have mild depression; 25-30 was considered as moderate depression, and a total score higher than 31 was diagnosed as severe depression (3, 10). The two latter groups were included in the study. Exclusion criteria were pregnancy and patients who were at high risk for suicide. Suicidal risk was determined through taking a comprehensive history by psychiatrist; patients who had suicidal idea and or plan were simply excluded from the study. All eligible patients were asked to give informed consent to participate in the study and to the collection of information regarding their health and wellbeing via a short questionnaire before experiment, and then at 4 week and 8 week after beginning of the study. Patients who consented to participate in the study were randomly assigned to experiment treatment (*Lavandula infusion*) or standard treatment groups.

### 3.4. Intervention

In standard treatment group, patients were ordered to use 20 mg Citalopram (Amin. Co, Iran) twice a day. In experimental treatment group, the patients took the same dose of Citalopram in addition to two cups of Lavandula Angustifilia infusion with sugar made by adding five grams of dried shoots of the herb to boiling water for duration of 8 weeks (11). Herbs had already weighted into 5 grams packages by the study coordinator. Patients were completely instructed to make and use this infusion properly and avoid using any other herbs during the study period. During the study time also the main reviewer using weekly phone calls to check whether they use the drug properly or not, likewise the patients were asked about any discomfort or complaint during the use of Lavandula Angustofolia, has followed them. Patients were withheld from the study if experienced any discomfort or inconvenience during use of the drug or more importantly if presented with suicidal idea at any point.

Patients were instructed to use this infusion and avoid using any other medications and herbs during the study period. Any modification in the protocol leads to excluding the patient from the study.

### 3.5. Outcomes Measure and Monitoring

The questionnaire was completed for each patient in a setting of an organized interview by the study coordinator, who was a trained medical intern, at the beginning of the study (before experiment), 4 weeks and 8 weeks after the beginning of the experiment. All patients were also received a form containing fourteen questions and answers about drug side effects such as confusion, increase or decrease of appetite, dry mouth, diarrhea and the other known side effects during the first visit. All the patients were biweekly visited by a psychiatrist. In addition, patients in the experimental treatment group were also weekly telephoned by the study coordinator to assess for drug side effects, depression signs and symptoms as well as suicide risk. In cases with a high risk for suicide, the patients were immediately referred to psychiatry hospital to perform the appropriate treatment plan.

### 3.6. Statistical Analysis

Data analysis was performed using SPSS 11, the statistical test including t-student test, t-pair test, and Chi Square were performed.

## 4. Results

100 patients were assessed for eligibility to enter the study and randomized into experimental treatment group and standard treatment groups. Each group contained 50 patients. Of 50 patients in the experimental treatment group, 3 patients lost to follow up while 7 patients discontinued intervention. Among standard treatment group, 10 patients lost to follow up, this higher number of loss in follow up in standard treatment group is maybe due to the lack of weekly telephones in this group. Finally 40 patients in each group were analysed successfully. Age range in the study population was between 17 to 74 years in which mean age in experimental treatment group and standard treatment group was 40.6 ± 13.6 and 40 ± 13 respectively. The experimental treatment group included 16 men while the standard treatment group contained 13. In terms of educational level, 55 patients were under diploma, 17 had diploma, 3 had an associate degree and 5 had a bachelor degree. Sixty two patients were married and eighteen were single. Forty two patients were living in rural areas where thirty eight were urban residents (Table 1). Mean depression score at the beginning of the study was 21.6 ± 4.9 in the standard treatment group and 22 ± 4.4 in the experimental treatment group. Statistical analysis showed no significant different between the two groups regarding demographic data and depression score before the intervention (Table 2). At the study onset, most patients in two groups had mild depression. Fisher's exact test indicated that the distribution of the severity of depression in the two groups does not have a significant difference. On the other hand, t­student test showed that mean depression.

**Table 1.** Baseline Demographic and Clinical Characteristics

	Intervention(Lavandula angustifilia, N = 40	Control (placebo), N = 40	P value
**Age**	Mean: 40.6	Mean: 40	P = 0.83
**Sex**			P = 0.49
Male	32.55	40%	
Female	67.5%	60%	
**Education**			P = 0.28
Under diploma	62.5%	75%	
Diploma	25%	17.5%	
University degree	12.5%	7.5%	
**Inhabitants**			P = 0.073
Urban	57.5%	37.5%	
Rural	42.5%	62.5%	
**Marital status**			P = 0.1
Married	85%	70%	
Single	15%	30%	

**Table 2.** Mean Depression Score before, After 4 and 8 Weeks of Intervention in Standard and Experimental Treatment Group

Group Time	Standard treatment group N = 40	Experimental treatment group N = 40	P value
**Before intervention**	21.6 ± 4.9	22 ± 4.4	P = 0.68
**After 4 weeks**	17.5 ± 3.5	15.2 ± 3.6	0.05 >
**After 8 weeks**	16.8 ± 4.6	14.8 ± 4	0.01 >

Score in four and eight weeks of the intervention was significantly lower in the experimental treatment group in comparison to the standard treatment group (P < 0.05) (see Table 2). In terms of patients satisfaction, In experimental treatment group, 25 patients were totally satisfied with the treatment and 9 were partially satisfied; however, in the standard treatment group, 19 patients were totally satisfied with the treatment and 13 were partially satisfied. Fisher's exact test showed no statistically significant difference in distribution of treatment satisfaction between the two groups. The most common adverse reactions in the experimental treatment group were Nausea (12.8%) and confusion(10%). In comparison, the most common side effects in standard treatment group were dry mouth (8.9%) and confusion (8%). There was no significant difference between the two groups regarding the side effects.

## 5. Discussion

The results of this study indicated that patients had no significant difference in terms of severity of the depression at the beginning. On the next follow ups (4 weeks and 8 weeks later), mean depression score in the standard treatment group who received only Citalopram was significantly higher than the experimental treatment group who consumed Lavandula infusion in addition to the same dose of Citalopram. Nowadays complementary therapies have become very popular and psychiatric disorders such as anxiety and depression are among the most common indications for using herbal remedies. Many depressed patients prefer to use complementary therapies such as herbal remedies; however, people's reasons for using these therapies are complicated and not clearly understood (12). Some reports have shown that many patients with psychological problems have used the beneficial effects of herbal remedies for their medical and somatic disorders including somatic disorders in depressed patients (5).

Akhunzade (2003) compared the effect of Lavandula Tincture and Imipramine in the treatment of mild to moderate depression. Their results indicated that the combination of Imipramine and Lavandula Tincture was significantly more effective than Imipramine alone. According to their findings, Lavandula Tincture might be of therapeutic value in treatment of mild to moderate depression (3). Our results which are in consistence with their study showing that after four weeks of intervention, patients had a significantly better condition regarding the depression score. It must be mentioned that it is not yet clear that Lavandula has a synergic or additive effect with citalopram and this must be taken into consideration in future researches. Effects of Lavandula on depression can be explained considering its multiple chemical constituents and their effect on variety of neurotransmitters involved in pathophysiology of depression. In different studies, the effects of this herb on Gamma Amino Bootiric Acid (GABA) has been proved which due to the role of GABA in mood disorders, therapeutic effects of the herb can be attributed to its effect on this neurotransmitter (2). On the other hand, it contains flavnoids which can effect on benzodiazepine receptors (13). Presence of multiple chemical constitutes such as monoterpenes and sesquiterpene including linalool, linalyl acetate and flavnoids such as L*. uteolin* in the other species of this plant (*L. vera*) reinforces the possibility of its effect on different parts of the central nervous system (14, 15). Shaw et al (2007) showed that the effects of Lavandula on rat are the same as chlordizpoxide (16)

Linalool in the plant is effective on Noradrenalin and dopamine level and increases them which might be another possible mechanism of the herb's anti-depressant effects (17). Rapid therapeutic effects of Lavandula can be attributed to the same mechanism of antidepressant effect of Hypericum (18). It is believed that Lavandula performs its psychological effects by affecting on limbic system particularly amygdala and hippocampus. Mechanism of action in cellular level is not totally clear, but some studies have suggested that its function is similar to benzodiazepines which cause the increment of GABA in amygdale (19). Re et al. (2000) also found that linalool in the herb can inhibit the release of acetylcholine and alter the function of the ionic canal in neuromuscular junction. Linalyl acetate has a narcotic function and linalool also acts as a sedative. This function can explain the traditionally use of this herb as a narcotic. Short term administration of this herb (for two weeks) has been shown to cause an improvement in night sleep condition and decreases demand for narcotic peels which in consistent with our findings that indicates the rapid effects of Lavandula (20). Walsh and Wilson (1999) in their investigation found that Lavandula and some other herbs can improve mood disorders and decrease psychological problems in patients (21). Massage therapy with Lavandula essential oil also has been effective in treatment of depression and sleep disorders in patients suffering from cancer (22). In some other studies, Jasmine herbal tea has shown relaxant and mood effective properties and linalool has been found as its major chemical constituent responsible for these effects. Anti-depressants effects of Lavandula infusion might also be attributed to the present of this chemical agent in its combination (23).

According to our results and considering the fact that the beginning of treatment response in anti-depressant drugs is 4­6 weeks (24), Lavandula might be used at the beginning as a complementary treatment to improve therapeutic effects because this improvement at the beginning can encourage the patients to continue the medication and increase patient satisfaction and compliance. One problem with herbs particularly in case of Lavandula is their appearance, odor, taste which might be a cause of nausea that was more frequently seen in the experimental group. In addition, the preparation process requiring instruments (for boiling water for instance) might decrease their usage in their present form of administration as herbal tea or infusion. Presenting these herbs in form of tablets or in standard packages in herbal remedy markets might be one possible solution. Also, using a placebo as a standard treatment group will strengthen the study which is one of our study limitations. Another limitation to our study is lack of blindness and this is because *Lavandula angustifilia* was not in the same shape as Citalopram in terms of appearance, therefore, both the study coordinator and the patient knew in which group they had been placed.

Considering the increasing popularity of herbal remedies among people as well as anti-depressant and relaxant effects of *Lavandula angustifilia*, the results of this study suggest the possibility of using this herb in depressed patients in order to obtain higher early anti-depressant effect and also decrease side effects of chemical anti-depressant medications such as Citalopram.

## References

[1] Whooley MA, Simon GE (2000). Managing depression in medical outpatients.. N Engl J Med..

[2] Kaplan HI, Sadocks BJ (2008). Synopsis of psychiatry behavioral sciences: clinical psychiatry..

[3] Akhondzadeh S, Kashani L, Fotouhi A, Jarvandi S, Mobaseri M, Moin M (2003). Comparison of Lavandula angustifolia Mill. tincture and imipramine in the treatment of mild to moderate depression: a double-blind, randomized trial.. Prog Neuro-Psychoph..

[4] Mann JJ (2005). The medical management of depression.. N Engl J Med..

[5] Akhondzadeh Sh, Maleki J (2006). Herbal medicines in the treatment of psychiatric and neurological disorders.. Iran J Psychiat..

[6] Linde K, Berner MM, Kriston L (2008). St John's wort for major depression.. Cochrane Database Syst Rev..

[7] Denner SS (2009). Lavandula angustifolia Miller: English lavender.. Holist Nurs Pract..

[8] Graham PH, Browne L, Cox H, Graham J (2003). Inhalation aromatherapy during radiotherapy: results of a placebo-controlled double-blind randomized trial.. J Clin Oncol..

[9] Lehrner J, Marwinski G, Lehr S, Johren P, Deecke L (2005). Ambient odors of orange and lavender reduce anxiety and improve mood in a dental office.. Physiol Behav..

[10] Maleki M, Javidi Z, Kiafar B, Saadatian V, Saremi AK (2005). Prevalence of depression in vitiligo patients.. QJ Fundamentals Mental Health..

[11] Sajadi E (2003). Lavandula In Medicinal plant pharmacopeia..

[12] Ernst Edzard (2007). Herbal remedies for depression and anxiety.. Advances in Psychiatric Treatment..

[13] Salah SM, Jager AK (2005). Two flavonoids from Artemisia herba-alba Asso with in vitro GABAA-benzodiazepine receptor activity.. J Ethnopharmacol..

[14] Gabrieli C, Kokkalou E (2003). A new acetylated glucoside of luteolin and two flavone glucosides from Lavandula stoechas ssp. stoechas.. Die Pharmazie-An International Journal of Pharmaceutical Sciences..

[15] Hajhashemi Valiollah, Ghannadi Alireza, Sharif Badie (2003). Anti-inflammatory and analgesic properties of the leaf extracts and essential oil of Lavandula angustifolia Mill.. J Ethnopharmacol..

[16] Shaw D, Annett JM, Doherty B, Leslie JC (2007). Anxiolytic effects of lavender oil inhalation on open-field behaviour in rats.. Phytomedicine..

[17] Yamada K, Mimaki Y, Sashida Y (2005). Effects of inhaling the vapor of Lavandula burnatii super-derived essential oil and linalool on plasma adrenocorticotropic hormone (ACTH), catecholamine and gonadotropin levels in experimental menopausal female rats.. Biol Pharm Bull..

[18] Ruedeberg C, Wiesmann UN, Brattstroem A, Honegger UE (2010). Hypericum perforatum L. (St John's wort) extract Ze 117 inhibits dopamine re-uptake in rat striatal brain slices. An implication for use in smoking cessation treatment?. Phytother Res..

[19] Cavanagh HM, Wilkinson JM (2002). Biological activities of lavender essential oil.. Phytother Res..

[20] Re L, Barocci S, Sonnino S, Mencarelli A, Vivani C, Paolucci G (2000). Linalool modifies the nicotinic receptor-ion channel kinetics at the mouse neuromuscular junction.. Pharmacol Res..

[21] Walsh E, Wilson C (1999). Complementary therapies in long-stay neurology in-patient settings.. Nurs Stand..

[22] Chang SY (2008). [Effects of aroma hand massage on pain, state anxiety and depression in hospice patients with terminal cancer].. Taehan Kanho Hakhoe Chi..

[23] Kuroda K, Inoue N, Ito Y, Kubota K, Sugimoto A, Kakuda T (2005). Sedative effects of the jasmine tea odor and (R)-(-)-linalool, one of its major odor components, on autonomic nerve activity and mood states.. Eur J Appl Physiol..

[24] Schulberg HerbertC, Raue PatrickJ, Rollman BruceL (2002). The effectiveness of psychotherapy in treating depressive disorders in primary care practice: clinical and cost perspectives.. General hospital psychiatry..

